# Improving adherence to an online intervention for low mood by a virtual coach or personalized motivational feedback messages: A three-arm pilot randomized controlled trial

**DOI:** 10.1016/j.invent.2025.100900

**Published:** 2025-12-26

**Authors:** Khadicha Amarti, Marketa Ciharova, Simon Provoost, Mieke H.J. Schulte, Annet Kleiboer, Ali el Hassouni, Gonçalo Gonçalves, Heleen Riper

**Affiliations:** a Department of Clinical, Neuro- and Developmental Psychology, Clinical Psychology Section, VU University, Amsterdam Public Health Research Institute, Amsterdam, the Netherlands; b Centre for Substance Use & Addiction Research (CESAR), Department of Psychology, Education and Child Studies, Erasmus University Rotterdam, Rotterdam, the Netherlands; c Department of Computer Science, Vrije Universiteit Amsterdam, Amsterdam, the Netherlands; d Mobiquity Inc., Data Science and Analytics, Amsterdam, the Netherlands; e Institute for Systems and Computer Engineering, Technology and Science, Porto, Portugal; f Department of Psychiatry, Amsterdam UMC, Location VU University Medical Centre, Amsterdam Public Health Research Institute, Amsterdam, the Netherlands

**Keywords:** Behavioural activation, Adherence, Motivational support, Personalization, Virtual coach, Low mood

## Abstract

**Background:**

Online psychological interventions like behavioural activation (BA) can be provided with or without human support. Unguided online interventions require no human contact and are therefore easier to implement on a large scale than guided interventions. However, effectiveness and adherence rates to these interventions are generally lower. One way to increase adherence to unguided online interventions is to offer automated motivational support.

**Objective:**

This pilot randomized controlled trial (RCT) examined whether adherence to unguided online BA for low mood could be improved by adding automated support in the form of smartphone-delivered personalized motivational messages or a motivational virtual coach.

**Methods:**

A three-arm pilot RCT (*n* = 106) was conducted that compared an online intervention delivered with automated motivational support by a virtual coach (*n* = 35), or by automated personalized messages on their smartphone (*n* = 35), to the same intervention without support (control condition; *n* = 36). The primary outcome was level of adherence, operationalized as (1) the number of webpages of the intervention visited, and (2) the number of mood ratings completed on the smartphone application, both retrieved from participants' logfiles. Secondary outcomes were satisfaction with the intervention (CSQ-I), usability (SUS) depression scores (HADS), and motivation for treatment (SMFL), measured through online questionnaires administered at baseline or after 4 weeks.

**Results:**

Adherence was moderate overall, with participants visiting on average 23 pages of 55 webpages and completing on average 50 of 84 requested mood ratings. No evidence for differences in adherence rates were observed between the intervention conditions and the control condition. Satisfaction with the intervention was moderate to high. Usability scores were below the desirable threshold of 68. Depression symptoms did not change significantly across all participants (*p* = .053). No significant changes in motivation were found over time or between groups.

**Conclusions:**

Adding automated support to unguided online BA for depression did not improve overall adherence. The limited effectiveness may reflect a misalignment between the motivational strategies and the needs of the target population, who reported mild symptoms and high intrinsic motivation. The findings highlight the need to further improve both the quality of automated support and the usability of online platforms. Future research should explore additional adherence-related factors and investigate how personalization can better address different symptom severities in unguided mental health interventions.

**Trial registration:**

International Clinical Trials Registry Platform: trialsearch.who.int/Trial2.aspx?TrialID=NL8110.

## Highlights

• Pilot RCT tested automated motivational support to improve adherence in unguided online BA for low mood.
• Compared personalized motivational messages and a virtual coach to no support
• Automated support did not significantly improve adherence or depression outcomes.
• Findings suggest need to refine motivational strategies and improve platform usability.
• Results inform future personalization of unguided digital mental health interventions.

## 1 Introduction

Unguided internet-based interventions based on cognitive behavioural principles, such as behavioural activation (BA), offer a scalable and accessible approach to dealing with mild depressive symptoms (Karyotaki et al., 2021; Yasukawa et al., 2024). However, a limitation of unguided interventions is low adherence, which can reduce the potential benefit of such interventions (van Ballegooijen et al., 2014; Karyotaki et al., 2015). Adherence, defined as the degree to which individuals engage with and complete the intervention (Christensen et al., 2009; Donkin et al., 2011), is linked to improved clinical outcome (van Ballegooijen et al., 2014; Forbes et al., 2023; Lüdtke et al., 2018). One commonly proposed solution to improve adherence in online mental health interventions is the use of human support (Borghouts et al., 2021; Jelinek et al., 2023). Guided internet-based interventions generally demonstrates higher adherence and effectiveness compared to unguided formats (Leung et al., 2022; Lundström et al., 2022; Karyotaki et al., 2021). However, the limited availability of trained professionals poses challenges to the scalability of guided interventions and leads to increased costs (Patel et al., 2018; Karyotaki et al., 2017). Therefore, it is imperative to identify solutions to improve adherence in interventions delivered without human support.

Motivation to participate in an intervention is an important factor influencing adherence rates; individuals that are motivated are more likely to initiate and maintain behaviour that supports their mental well-being (Hanano et al., 2024; Jardine et al., 2022). Research has shown that individuals' motivation can be improved with the use of automated motivational strategies which in turn improve adherence to online interventions (Lu et al., 2025; Jelinek et al., 2023; Donkin and Glozier, 2012). There are different strategies to increase motivation for taking part in online interventions, such as automated messages, chatbots, virtual coaches, interactive self-help modules, and gamification (Six et al., 2021; Saad et al., 2021; Danieli et al., 2021; Schure et al., 2019). Motivational strategies can be applied both before and during an intervention to encourage individuals to reflect on their goals, improve self-efficacy, and provide feelings of empathy and support (Schunk and DiBenedetto, 2020). By helping individuals recognise what they can achieve and reinforcing progress, motivation can play a significant role in sustaining engagement in the intervention. To address the challenge of low adherence in unguided BA interventions, the use of automated motivational support could be a potential solution.

One approach to improve motivation for an intervention is motivational interviewing (MI; Harkin et al., 2023; Marker and Norton, 2018). The cornerstone of MI is to help individuals resolve ambivalence about change by strengthening their intrinsic motivation and promoting behavioural change, while doing so in a compassionate, empathic, and non-judgemental manner (Miller and Rollnick, 2002). MI is a well-established conversational therapeutic technique that may effectively improve adherence to an intervention and is increasingly? applied in online interventions. For instance, Titov et al. (2010) integrated MI strategies into an unguided internet-based cognitive behavioural therapy (iCBT) program for social phobia, to help participants explore their ambivalence about change. Participants receiving this additional motivational support demonstrated higher completion rates (75 %) compared to those in the standard iCBT group (56 %). Furthermore, a recent RCT among employees with subthreshold depression demonstrated that adding an automated chatbot to an iCBT program significantly increased completion rates from 19.2 % to 34.8 % (Yasukawa et al., 2024).

One way to incorporate MI techniques to unguided online interventions is with an embodied conversational agent (ECA) in the form of a virtual coach. Virtual coaches are computer-generated characters that simulate verbal, textual and nonverbal cues that are essential in human face-to-face communication (Provoost et al., 2017). A virtual coach can simulate several human support factors, such as motivational interviewing techniques, feedback to CBT exercises and empathic communication (Ruttkay et al., 2004). For example, the virtual coach can pose reflective questions to increase motivation and/or give positive reinforcement when activities are completed. There is growing evidence supporting the role of conversational agents in mental health interventions, as described in the systematic reviews by Vaidyam et al. (2019) and Gaffney et al. (2019), which highlights their potential as solutions to improve adherence to an intervention.

Another strategy to improve motivation in an online intervention is automated personalized messaging. This strategy involves delivering tailored motivational messages to participants, aiming to improve their adherence to the intervention. Supporting this approach, Hornstein et al. (2023) conducted a systematic review highlighting that automated personalization strategies, such as targeted communication and tailored content, can improve adherence. They found that personalization often involved tailoring communication style and modifying content according to decision rules and/or user input. Hochberg et al. (2016) demonstrated that personalized messages, optimized through reinforcement learning (RL), effectively increased physical activity among individuals with type 2-diabetes. In their study, messages were categorized into distinct types, and the algorithm learned which message type and timing worked best for each individual, based on ongoing feedback. Participants receiving RL-personalized messages showed significantly better adherence compared to those receiving standard messages. RL is a machine learning (ML) technique that learns how to make decisions based on feedback from its environment (Jayaraman et al., 2024). The types of messages that RL systems aim to optimize can reflect core therapeutic functions, such as promoting autonomy and validating an individual's emotions, similar to the therapist strategies identified in a study by Mol et al. (2018) on online feedback in blended therapy for depression. el Hassouni et al. (2022) designed a personalization architecture named pH-RL (personalization in e-Health with Reinforcement Learning) to improve internet del interventions through automated adaptive personalized messaging. By using reinforcement learning (RL) techniques, the system learns to select and deliver the most suitable personal motivational messages with the aim to increase participants' adherence to the intervention. This study found that RL techniques could effectively adapt to participants' behaviour and preferences and lead to improved adherence over time.

Both strategies aim to improve adherence, but they differ in mechanism and potential applicability. Virtual coaches provide human-like interaction, empathic feedback, and motivational support, which may be particularly beneficial for individuals with lower motivation or less experience with digital interventions. Personalized messages, in contrast, adapt to user behaviour and offer a highly scalable and flexible solution that may be sufficient for users who are already self-motivated. The relative effectiveness of each approach may therefore depend on participants characteristics, preferences and context.

In the current study, we conducted a pilot randomized controlled trial (RCT) to examine whether adherence to an unguided online BA intervention for low mood could be improved by adding automated motivational support. The primary aim was to compare adherence rates between a control condition receiving BA without additional support and two intervention conditions; one that received automated support from a virtual coach using MI techniques, and another that received personalized motivational messages via a smartphone application. Secondary aims were to assess the feasibility of the intervention in terms of user satisfaction and usability, and to explore changes in depressive symptoms. By focusing on adherence, this study aimed to provide insights into the potential of automated strategies to improve engagement in unguided online interventions for low mood.

## 2 Methods

### 2.1 Design

This study is a three-arm pilot RCT that compared an internet delivered BA intervention without support (control condition; *n* = 36) to the same intervention delivered with automated motivational support by a virtual coach (*n* = 35), or by automated personalized motivational messages on a smartphone (*n* = 35). The study was approved by the Medical Ethics Committee of the VU Medical Centre, Amsterdam (registration number 2019.388) and full details of the study have been published in the protocol paper (Provoost et al., 2020). The original protocol described a two-arm design; the personalized messages condition was added during the preparation phase to allow comparison of different forms of automated support. As a result, the analysis plan presented here differs from the preregistered protocol, and these modifications were made to accommodate the additional intervention arm.

### 2.2 Study population

Participants were recruited from the general population through social media (Facebook) and with the assistance of an online recruitment service (Link2Trials BV) between August 2021 and January 2022. Link2Trials is a platform that connects researchers with volunteers interested in participating in studies, facilitating recruitment of a broad community sample. While no specific quotas were applied, recruitment through this channel aimed to reach a diverse adult population in terms of age, gender, and education. Participants were included if they (1) answered positively on the question “do you want to improve your mood?” and (2) were 18 years or older. Participants were excluded if they (1) did not have a computer with internet access, (2) did not have a smartphone, (3) had moderate to severe depression (as measured with the PHQ-9; a score of 15 or higher), or (4) were identified as being at risk for suicide (as measured with item 9 of the PHQ-9 with a score of 1 or higher indicating suicidal ideation). We specifically targeted adults from the general population who indicated a desire to improve their mood. This allowed us to examine adherence and feasibility of a brief, unguided program in a broad sample before testing in a clinical population, providing important insights into engagement patterns among motivated users. Excluded participants received an email detailing the reason for their exclusion. If participants were excluded because they had moderately to severe depression, they were advised to contact their general practitioner; if participants were identified as being at risk for suicide they were advised to contact a national help crisis line for people at risk of suicide (www.113.nl).

### 2.3 Randomization

Participants were randomly assigned to one of the three conditions using Castor EDC (Castor Research Inc.). The allocation was determined using a 1:1:1 ratio, and randomization was conducted through an automated computer-generated block randomization table featuring variable block sizes, assuring allocation concealment. Group allocation was not blinded to the participants, because a description of the study's research aims was provided in the informed consent letter.

### 2.4 Sample size

Our main aim was to explore the potential of increasing adherence to an online intervention by means of automated support. As this study tests new add-on intervention components our calculation is based on the recommendations of Teare et al. (2014), which is a sample size of *n* = 35 per group for explorative studies. With three groups, we planned on recruiting 105 participants.

### 2.5 Study procedure

People who were interested in participating in the pilot study could register through a dedicated research website. When registered, potential participants received an information letter and a printed informed consent form by post. Those who were willing to take part in the study were asked to sign and return the informed consent form by post. The procedure of consent via the post was required by the METC, which mandated written informed consent for ethical approval. When the signed informed consent form was received, participants received a link to the online screening questionnaire (PHQ-9) through Castor. Eligible candidates received a link to the baseline (T0) questionnaire. Once the baseline questionnaire was filled out, participants were randomized to one of the three conditions. The login details for platform and a manual on the use of the platform was shared with the participants through secured email. The post-treatment assessment (T1) was sent to the participants four weeks after the baseline assessment (T0). Participants received a 30-euro voucher after they completed the post assessment questionnaire.

### 2.6 Intervention

The intervention used in this pilot RCT was *Moodbuster Lite*, a four-week online BA course aimed at improving low mood, built in the platform Moodbuster. Moodbuster is an online platform designed to provide evidence-based psychological interventions for mental health conditions. The platform consists of a web portal for patients and practitioners and a smartphone application in which mood and other activities can be measured. The intervention on the web-based platform is a shortened version of the Moodbuster for Depression intervention (Warmerdam et al., 2012). *Moodbuster Lite* focuses specifically on behavioural activation (Lewinsohn et al., 1976) to encourage positive behavioural change, because behavioural activation therapy alone is as effective as full cognitive behavioural therapy for treating depression (Ciharova et al., 2021). As this was the first evaluation of the Lite version, the study also served to examine its feasibility.

The *Moodbuster Lite* course consists of three online lessons: (1) Introduction (to the platform) (2) Psychoeducation, and (3) Pleasurable Activities. The Introduction module included up to 22 pages, the Psychoeducation module up to 13 pages, and the Pleasurable Activities module up to 20 pages. In the virtual coach condition, additional pages were included that featured exercises of feedback within each module. The smartphone application, designed for both Android and iOS, prompts participants with a request to rate their mood three times a day at random times on the app. Participants were advised to complete the intervention over a time span of four weeks. There was no fixed or mandatory order in which participants had to complete the modules, allowing users some flexibility in how they navigated the course. However, within each module, participants could not proceed to the next page or section without first completing the required exercises.

#### 2.6.1 Intervention with virtual coach

**Fig. 1:**
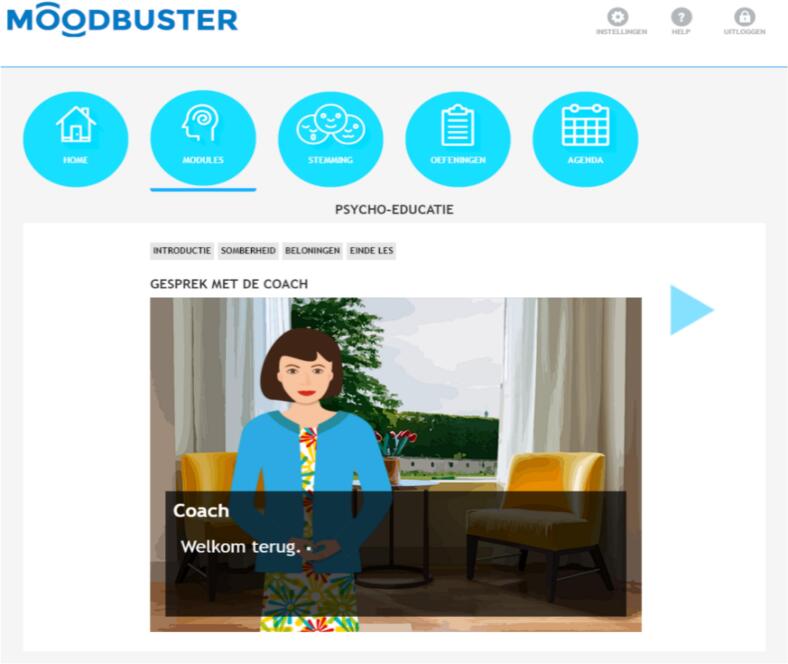
The virtual coach embedded in the Moodbuster Lite platform.

As part of this study, a virtual coach (Provoost et al., 2020) was embedded in the platform for participants who were randomized to the condition with virtual coach support. The virtual coach was created in Tyranobuilder, a JavaScript-based software tool for creating visual novels that can be exported in a browser-format that allows them to be embedded in web pages (Fig. 1). The virtual coach gave personalized motivational feedback on the exercises at the beginning and end of every lesson and halfway through lesson 3. The written conversations with the virtual coach were developed in collaboration with a licensed therapist (Mol et al., 2018) and motivational interviewing principles (Rollnick et al., 2008). The focus on motivational interviewing was to improve an individual's willingness to change their behaviour and build their confidence, both of which are important for promoting a person's ‘readiness’ to change. The intervention assesses willingness to change in Lesson 1, using importance and confidence ruler exercises (Provoost et al., 2020). If either is low, the virtual coach provides tailored exercises to emphasize the need for change or boost self-efficacy. The virtual coach was accessible only after a participant logged into the intervention platform at least once, meaning that participants who never initiated the program did not receive any coaching.

#### 2.6.2 Intervention with personalized messages

The personalized messages were automatically sent to the smartphone application of the participants who were randomized to the condition with personalized messages. The messages were personalized using the pH-RL architecture developed by el Hassouni et al. (2022). This reinforcement learning framework initially uses baseline characteristics—such as gender, age, and participant preferences for types of motivational messages (see below) — to tailor the personalized messages. As the study progressed, the algorithm incorporated real-time data, including mood ratings and user interactions, to further improve message personalization. The types of the motivational messages were created according to the most common human support messages in guided online interventions for subclinical depression, which involve information, encouragement, and affirmation (Mol et al., 2018). *Informing behaviour* refers to messages aiming to inform or refer to different functionalities on the online platform, such as informing about the next session (“You can now proceed with session 5.”). *Encouraging behaviour* aims to motivate and encourage past and future behaviour of the participant by, for example, encouraging something the participant is planning to do (“Continue to keep up this good work! Good luck!”). Finally, *affirming behaviour* gives attention, recognizes and shows interest in thoughts, emotions and behaviours of the participant and considered them valid, such as validating what the participant has shared in the intervention (“That must be very difficult for you to see that you are struggling with it.”). The pH-RL system used clustering techniques to group users based on behavioural patterns. This approach allowed the system to select the most suitable message type — affirming, informative, or encouraging — for each participant at different times, depending on their previous adherence to the mood rating after such messages. Participants needed to have downloaded the smartphone application to receive and view the messages. Therefore, participants who did not initiate the intervention did not receive any personalized messages.

### 2.7 Assessments

All measures, except for adherence, were assessed through web-based self-report questionnaires. Adherence was measured through logfile analysis. Logfile analysis is a method used to monitor and evaluate user interactions with online interventions through automatically recorded data (Christensen et al., 2009; Oehler et al., 2021). These logs contain detailed information such as login frequency, time spent on the platform, number of modules completed, and activities performed. Analysing these logs allows researchers to assess adherence to the intervention. Demographic information was collected at baseline and included age, gender, educational level, employment status, nationality and relationship status.

#### 2.7.1 Primary outcome measures

##### 2.7.1.1 Adherence

The primary outcome is level of adherence, defined as the extent to which individuals were exposed to the content of the intervention (Christensen et al., 2009). This was measured in two ways using logfile analysis: the total number of pages visited for more than fifteen seconds and the number of filled in mood ratings on the smartphone application. Spending fifteen seconds or more on a page was applied as cutoff for the potential to have read and engage with the content. This threshold was selected based on examination of the page visit data, which showed many very brief visits indicative of participants quickly clicking through pages. The cutoff helped ensure inclusion of participants likely to have meaningfully engaged with the content.

##### 2.7.1.2 Reasons for non-adherence

Reasons listed for non-adherence were assessed with one question: “Did you finish the Moodbuster Lite course?”. If their response was negative, they were asked to provide a reason for not having completed the intervention.

#### 2.7.2 Secondary outcome measures

##### 2.7.2.1 Motivation

Motivation for taking part in the intervention was measured by the Short Motivation Feedback List (SMFL; Jochems et al., 2014). The SMFL is based on self-determination theory (Ryan and Deci, 2008). It consists of eight 10-point Likert-scale items ranging from 0 (completely disagree) to 10 (completely agree), designed to capture the level and type (external, introjected, or identified) of a patient's treatment motivation. Identified motivation reflects participation based on personal values or perceived importance, introjected motivation refers to internal pressures such as guilt or the need to meet expectations, and external motivation is driven by outside demands or incentives (Jochems et al., 2014). Total scores range from 0 to 80, with higher scores indicating higher motivation to start the course. The three subscales have the following ranges: identified motivation (3 items; range 0–30), introjected motivation (2 items; range 0–20), and external motivation (3 items; range 0–30). The SMFL has demonstrated good reliability, with Cronbach's alpha values ranging from 0.81 to 0.93 (Jochems et al., 2014). There are two different versions: pre- and post-intervention version. The pre-intervention version was administered at baseline (T_0_) and the post-intervention version after four weeks (T_1_).

##### 2.7.2.2 System usability

Usability of the intervention was assessed by the System Usability Scale (SUS; Brooke, 1995). The SUS consists of ten 5-point Likert-scale items with response options ranging from 0 (strongly disagree) to 4 (strongly agree). Total scores are converted to a scale ranging from 0 to 100, where higher scores are indicative of higher platform usability. The SUS is considered a reliable instrument with scores higher than 68 indicating “good” usability (Cronbach's α = 0.90; Bangor et al., 2008, Lewis et al., 2015).

##### 2.7.2.3 User satisfaction

User satisfaction of the intervention was assessed by the Client Satisfaction Questionnaire for internet-based interventions (CSQ-I; Boß et al., 2016), which is an adaptation of the original CSQ (Larsen et al., 1979). The CSQ-I is composed of eight 4-point Likert-scale items with response options ranging from “does not apply to me” to “applies to me.” Total scores range from 8 to 32, with higher scores indicating greater client satisfaction. The CSQ-I has been found to be a reliable instrument (Cronbach's α = 0.87; Boß et al., 2016).

##### 2.7.2.4 Depression severity

Depression severity was assessed with the Depression subscale of the Hospital Anxiety and Depression Scale (HADS-D; Snaith and Zigmond, 1986). The HADS-D comprises seven items, each assessed on a 4-point scale with response options ranging from 0 to 3; a cut-off score of 8 indicates relevant symptoms for depression (Zigmond and Snaith, 1983). Total scores range from 0 to 21, and higher scores indicate more severe depression symptoms. The HADS-D has shown to be a reliable instrument (Cronbach's α: 0.93; Kwan et al., 2019).

#### 2.7.3 Other measures

Screening for mental health issues was conducted before group allocation (T − 1) using the Patient Health Questionnaire-9 (PHQ-9; Kroenke et al., 2001), to detect individuals with moderate to severe depressive symptoms. The PHQ-9 includes nine items scored from 0 (“not at all”) to 3 (“nearly every day”), resulting in a total score ranging from 0 to 27. Scores above 14 indicate moderate to severe depression and were used as part of the exclusion criteria. The PHQ-9 has demonstrated good psychometric properties in previous research (Wittkampf et al., 2007).

### 2.8 Analyses

Data analyses were performed using IBM SPSS version 27 (IBM Corp, 2020). Because the primary outcome was adherence to the digital intervention, we used a per-protocol analytic approach, meaning that analyses focused on participants who were randomized and accessed the intervention at least once. Including participants that did not at least start the intervention would not provide meaningful data on adherence, as they did not have the opportunity to adhere to the intervention. Missing data were not imputed; analyses were conducted using available data only, following an available-case (listwise deletion) approach. As adherence data were complete for participants who started the intervention, this approach ensured that adherence outcomes reflected actual user behaviour. In addition, we conducted supplementary analysis including the full randomized sample, with non-initiators scores as zero. The significance level used was *p* < .05. As this was a pilot trial with a small sample, group comparisons were considered exploratory. Descriptive analyses were used to describe demographic characteristics and to assess secondary outcomes, including depressive symptoms, motivation and feasibility of the “Moodbuster” platform in terms of usability and satisfaction at post-assessment. To assess the difference in the primary outcome, (i.e., adherence), between the intervention conditions and the control condition, four independent samples *t*-tests were conducted: one for each adherence measure (total pages visited and number of mood ratings) for each comparison (virtual coach vs. control, personalized messages vs. control).The dependent variable was the adherence measures (total pages visited and total mood ratings) and type of condition was the independent variable. Direct comparison between the two intervention conditions was not conducted, as the study was not powered for such comparisons and the primary objective was to evaluate the effect of each intervention condition relative to the control condition. The reasons for non-adherence that participants reported were analysed by thematically categorizing their open-ended responses using an inductive approach. Two authors (KA and MS) independently coded the responses, and discrepancies were resolved through discussion to reach consensus.

For the analysis of changes in the secondary outcomes over time between either of the intervention conditions (virtual coach or personalized messages) and the control condition, two repeated measures ANOVAs were conducted for depressive symptoms and two for motivation. The primary aim was to evaluate whether each automated support strategy improved outcomes relative to control. The dependent variable was the respective outcome (depression or motivation score), condition was the between-subjects factor, and time (T0, T1) was the within-subjects factor.

## 3 Results

### 3.1 Participants

**Fig. 2:**
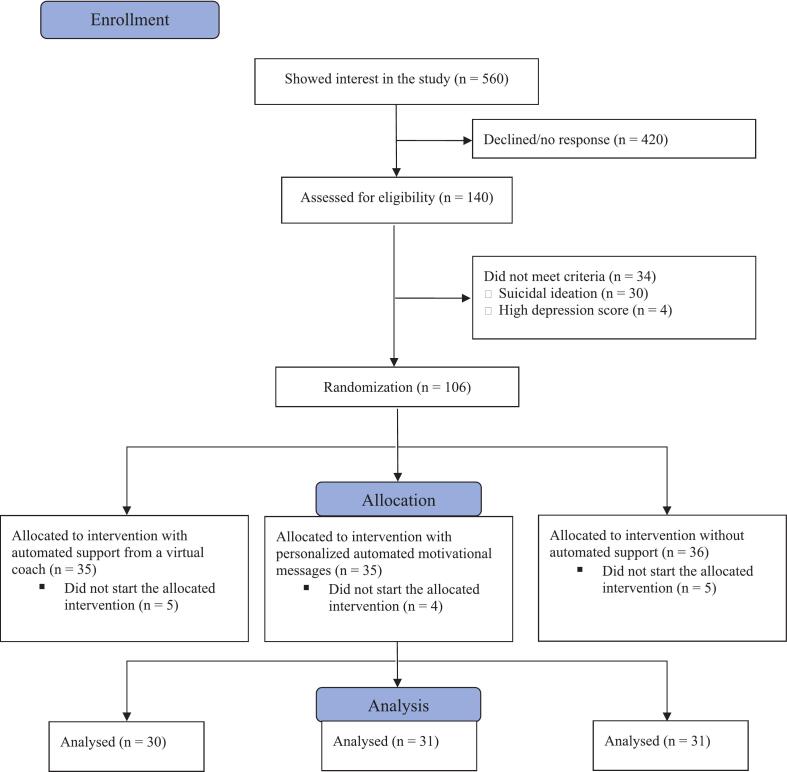
Flowchart.

Of the 560 people who showed interest in the study, 140 (25 %) were assessed for eligibility, and 106 (19 %) were randomized to one of the three conditions: virtual coach condition (*n* = 35), personalized messages condition (*n* = 35) or control condition (*n* = 36). The CONSORT flow diagram of the study is shown in Fig. 2. After four weeks, 87 % of the randomized participants (*n* = 92) completed the post-assessment questionnaires, representing a 13 % dropout rate (*n* = 14). All analyses reported in this manuscript were conducted with these 92 participants who started the intervention. All participants who started the intervention also completed the post-assessment.

Table 1 presents the characteristics of the participants. Of the 106 participants, 92 patients (87 %) started the intervention. Most participants were female (87 %), Dutch, highly educated and employed.

**Table 1:** Baseline sample characteristics (*N* = 92).

Participant characteristics	Virtual coach (*n* = 30)	Personalized messages (*n* = 31)	Control (*n* = 31)	Total (*n* = 92)
**Age (years), mean (SD)**	42.4 (13.67)	46.0 (15.0)	37.7 (13.57)	42.1 (14.36)
**Female, n (%)**	27 (90.0)	26 (83.9)	27 (87.1)	80 (87.0)
**Education, n (%)**^a^				
High school	1 (3.8)	2 (7.7)	0 (0)	3 (3.7)
University of Applied Science	12 (46.2)	14 (53.8)	9 (30.0)	35 (42.7)
University	13 (50.0)	10 (38.5)	21 (70.0)	44 (53.7)
**Dutch, n (%)**	23 (76.7)	26 (83.9)	30 (96.8)	79 (85.9)
**In a relationship, n (%)**	14 (46.7)	23 (74.2)	15 (48.4)	41 (44.6)
**Working, n (%)**	17 (65.4)	17 (63.0)	26 (86.7)	60 (72.3)
**Depression baseline score**^b^**, mean (SD)**	5.3 (3.4)	6.5 (2.9)	6.8 (3.3)	6.2 (3.2)
**Motivation baseline score**^c^**, mean (SD)**	29.2 (13.7)	32.4 (9.2)	32.8 (11.2)	31.4 (10.9)

a Ten participants did not fill in their education level.

b HADS-D: Hospital Anxiety and Depression Scale.

c SMFL: Short Motivation Feedback List.

### 3.2 Intervention adherence

Only the 92 participants who started the intervention were included in the analysis on intervention adherence. Across all three conditions, participants visited on average 23 pages (SD = 12.45; range 1–55) and completed an average of 50 mood ratings (SD = 49.89; range 0–386). The wide range in mood ratings is explained by one outlier who completed 386 mood ratings. Overall, nine participants (9.8 %) completed all modules, and 12 participants (13 %) completed at least 84 requested mood ratings. The descriptive data suggest a large variability in adherence to the intervention. As shown in Table 2, there was no significant difference between the virtual coach condition and control condition for both adherence measures. Similarly, there were no significant differences between the personalized messages condition and control condition for both adherence measures. Across conditions, effect sizes were small, indicating minimal practical differences in the adherence measures.

**Table 2:** Comparison of the adherence measures between virtual coach/control condition and between personalized messages/control condition (*N* = 92).

Adherence measures	Condition	*N*	*M*^a^	*SD*	(*t*)*df*	*p*	Cohen's d	95 % CI for *d*
Total pages visited (≥15 s)	Control	31	24.97	11.61				
	Virtual coach	30	23.13	13.16	−0.058 (59)	0.566	−0.015	[−0.65, 0.36]
	Personalized messages	31	20.58	12.57	1.43 (60)	0.159	0.036	[−0.14, 0.86]
Number of mood ratings	Control	31	52.40	32.46				
	Virtual coach	30	48.45	40.86	−0.041 (57)	0.682	−0.011	[−0.62, 0.40]
	Personalized messages	31	51.23	69.26	−0.37 (60)	0.488	−0.09	[−0.51, 0.49]

a Mean difference scores for the pages visited range from 0 to 55 and for the mood ratings from 0 to 84.

We additionally conducted adherence analysis including the full randomized sample (*N* = 106), with non-initiators obtaining a score of zero for both adherence measures. These results are presented in Annex 3. The pattern of findings was comparable to that in Table 2, showing variability in adherence scores but with lower means and no significant differences between the intervention conditions and control condition.

### 3.3 Reasons for non-adherence

In total 25 participants reported a reason at post-assessment for not completing the intervention. These are summarized in Table 3, organized into four categories. The most common reasons mentioned for not completing the intervention (40 % of the cases) were in the category “time-consuming”. Some participants found the platform difficult to use due to the complexity of the platform. One participant said, “*I didn't understand in what order I had to continue*”. The intervention being time-consuming was also a challenge, as one participant noted, “*I had a busy schedule and couldn't always focus on it*”. Additionally, some found the intervention confronting, such as one participant who said, “*The app kept reminding me that I could feel down, which made me feel worse*”.

**Table 3:** Reasons for non-adherence; not completing the intervention.

Category of reasons	Values, *n* = 25 (%)
Complexity of the platform	9 (36)
Confronting	2 (8)
Not helpful	4 (16)
Time-consuming	10 (40)

### 3.4 Feasibility analysis

Table 4 presents the results regarding system usability and intervention satisfaction. Participants in all conditions scored slightly below the desirable usability score of 68 or more for the interventions used. In all conditions, participants scored moderate on the CSQ-I measuring satisfaction with the intervention. There were no significant differences between either of the intervention conditions and the control condition for both feasibility measures (SUS and CSQ-I).

**Table 4:** Descriptive statistics of the SUS and CSQ-I.

Feasibility	Condition					
	Virtual coach *n* = 28		Personalized messages *n* = 29		Control *n* = 30	
	*M*	*SD*	*M*	*SD*	*M*	*SD*
SUS	66.07	21.26	63.28	20.93	67.5	17.14
CSQ-I	20.72	5.12	20.93	4.09	20.66	5.86

Note. Mean difference scores could theoretically range from 0 to 100 for the SUDS and 8–32 for the CSQ-I, with lower scores indicating lower usability and/or lower satisfaction/acceptance.

SUS: System Usability Scale.

CSQ-I: Client Satisfaction Questionnaire for Internet-based Interventions.

### 3.5 Effects of the intervention on motivation scores

Table 5 provides descriptive statistics for the motivation scores of each group at baseline and post-assessment. Results from the analyses are reported in Table 6. There were no significant main effects of time and group, and no significant interaction between time and group. Similarly, for the personalized messages vs control, there were no significant main effects of time and group, and no interaction effect. Descriptive statistics indicated that participants' scores on the “identified motivation” subscale were generally higher than the other two subscales at both baseline and post-assessment; no statistical comparisons were conducted between subscales.

**Table 5:** Descriptive statistics of motivation scores for each condition.

SMFL	Condition		
	Virtual coach *n* = 27	Personalized messages *n* = 27	Control *n* = 29
*Baseline assessment, M (SD)*			
Total motivation	28.56 (12.52)	32.63 (9.74)	32.41 (11.58)
External motivation	3.44 (4.97)	4.00 (3.93)	3.86 (4.41)
Introjected motivation	6.63 (5.31)	7.48 (3.79)	7.79 (4.44)
Identified motivation	18.48 (5.44)	21.15 (4.31)	20.76 (5.81)

*Post-assessment, M (SD)*			
Total motivation	28.15 (10.69)	30.56 (10.99)	30.41 (11.24)
External motivation	3.15 (4.21)	3.48 (3.75)	3.48 (3.75)
Introjected motivation	5.30 (4.82)	6.22 (4.17)	6.21 (5.39)
Identified motivation	19.70 (4.72)	20.21 (4.86)	20.72 (4.95)

**Table 6:** ANOVA of the sample for the motivation scores over time and group.

Comparison	Effect	F	*df*	*p*	η^2^ₚ
Virtual coach vs control	Time	0.930	1,54	.339	0.017
	Group	1.181	1,54	.282	0.021
	Time × group	0.407	1,54	.526	0.007
Personalized messages vs control	Time	2.316	1,54	.134	0.041
	Group	0.005	1,54	.945	0.000
	Time × group	0.001	1,54	.978	0.000

### 3.6 Effects of the intervention on depression scores

Across the total sample, we observed no significant reduction in depression symptoms at baseline (*M* = 6.20, *SD* = 3.26) and post-assessment (*M* = 5.60, *SD* = 3.54); *t* (86) = 1.96, *p* = .053, *95* *% CI* [−0.009, 1.204]. The effect size was small (Cohen's *d* = 0.21). Table 7 presents the descriptive statistics for each group for baseline and post-assessment. Table 8 presents the results of both analyses. For the comparison between virtual coach condition and control condition, there was no significant main effect of time and group, and no interaction effect of time x group. For the comparison between personalized messages condition and control condition there was a significant main effect of time, *F* (1, 57) = 5.72, *p* = .020, η^2^ₚ = 0.091, indicating that overall depression scores decreased from pre-test (*M* = 6.69, *SD* = 3.13) to post-test (*M* = 5.81, *SD* = 3.51). However, there was no main effect of group and the interaction between time and group was not significant.

**Table 7:** Descriptive statistics of depression scores for each condition.

HADS-D	Condition		
	Virtual coach *n* = 30	Personalized messages *n* = 31	Control *n* = 31
Baseline assessment, M (SD)	5.27 (3.42)	6.52 (2.85)	6.84 (3.34)
Post-assessment, M (SD)	5.14 (3.62)	5.90 (3.70)	5.73 (3.38)

**Table 8:** ANOVA of the sample for the depression scores over time and group.

Comparison	Effect	F	*df*	*p*	η^2^ₚ
Virtual coach vs control	Time	2.441	1,56	.124	0.042
	Group	2.048	1,56	.158	0.035
	Time × group	2.441	1,56	.124	0.042
Personalized messages vs control	Time	5.724	1,57	.020	0.091
	Group	0.026	1,57	.873	0.000
	Time × group	0.628	1,57	.432	0.011

## 4 Conclusion and discussion

### 4.1 Principal findings

This pilot randomized controlled trial aimed to examine whether automated motivational support could improve adherence to an unguided internet-based behavioural activation (BA) intervention for depressive symptoms. Participants were randomized to receive either (1) unguided BA with automated support from a virtual coach, (2) unguided BA with personalized automated messages, or (3) unguided BA alone. The results showed no meaningful differences in adherence between the intervention conditions and the control group, with adherence levels being moderate across all groups. Platform satisfaction was generally moderate to high, although usability ratings fell below the accepted threshold in all conditions. Participants demonstrated high levels of identified motivation at both baseline and follow-up, suggesting they recognized the importance of working on their well-being. However, motivation did not significantly change over time nor differ between groups. Similarly, the intervention had limited impact on depressive symptoms, with no notable differences between conditions.

### 4.2 Implications

Contrary to our hypothesis, automated motivational support did not improve adherence to the intervention. This finding contrasts with previous research that has demonstrated the potential of automated support mechanisms to improve adherence to online interventions (Borghouts et al., 2021; Jelinek et al., 2023). For example, Kelders et al. (2015) conducted a systematic review indicating that online interventions incorporating motivational design elements, such as reminders and tailored feedback, showed higher adherence rates compared to those without such features. Similarly, Schure et al. (2019) reported that participants that received standardized automated email reminders showed increased engagement in terms of module completion, in an online CBT program. However, our findings align with studies indicating that automated support alone may not be sufficient to improve adherence. In the systematic review of Groot et al. (2023) it was concluded that while unguided online interventions can positively impact mental well-being, maintaining adherence is a significant challenge. Ebert et al. (2018) highlighted that the absence of human support often leads to lower adherence rates in online unguided interventions, suggesting that automated support alone may not be sufficient. It should also be acknowledged that the current adherence measures capture only behaviour within the intervention and may underestimate participants' engagement with the intervention content in daily life. Individuals may have practiced behavioural activation techniques offline, which was not reflected in the log data or examined in the questionnaires.

Another important consideration is that the motivational strategies implemented may not have been insufficient in themselves, but rather that the target population was not suitable for these strategies. Participants' descriptive scores on the “identified motivation” subscale at both time points, suggest that motivation to work on one's well-being was already relatively high, leaving little room for improvement at post-assessment (Jochems et al., 2014). Therefore, the lack of observed effect may not reflect the ineffectiveness of the motivational support given, but rather the need for more personalized strategies that better align with participants' existing motivational level. Future iterations of the interventions could benefit from co-creation with end-users to ensure that both the virtual coach and the personalized messages are tailored to user's preferences, needs and motivational profiles. Furthermore, unguided intervention have generally been shown to be effective (Karyotaki et al., 2017), particularly in individuals with moderate to severe depressive symptoms. As participants in our sample reported mild to no depressive symptoms at baseline, the intervention may have offered limited added value for this group. In addition, the Moodbuster Lite program evaluated in this trial represented a brief, newly adapted version of the Moodbuster for Depression intervention. While this format was designed to explore adherence improvement and feasibility among individuals who want to improve their mood, it only included core components of behavioural activation (BA) and did not include cognitive therapy (CT). This abbreviated structure may partly explain the absence of significant change in depressive symptoms and relatively modest adherence observed. The shorter duration may have provided fewer opportunities for participants to engage meaningfully with the intervention. Furthermore, baseline depressive symptoms in our sample were relatively low, leaving limited room for improvement.

Previous research has also highlighted that usability issues, such as complex navigation or unclear instructions, can negatively affect adherence in online interventions (Borghouts et al., 2021; Beatty and Binnion, 2016). While participants reported moderate to high satisfaction with the intervention, usability was rated below the desirable threshold, which may have contributed to lower adherence. Although usability scores were below the standard acceptability threshold, this study did not assess the statistical association between usability and adherence outcomes. Nevertheless, this finding is consistent with studies showing that even when users perceive an intervention as valuable, moderate usability can hinder sustained use (e.g. Perski et al., 2017).

### 4.3 Strengths and limitations

To the best of our knowledge, this study is one of the first to investigate the effect of automated support on adherence in a three-armed pilot RCT, which allowed for a comparison of two types of automated support (virtual coach and automated messages) against a control condition. Furthermore, we incorporated motivational components in the intervention, which is more frequently seen in interventions for substance abuse (Schulte et al., 2022; Martens et al., 2013; Ray et al., 2014) and less in interventions for low mood or depression. Lastly, including feasibility measures, such as usability and satisfaction, made it possible to gain insight into user experience with the platform and identify potential barriers to adherence.

However, several limitations should be noted. First, this was an explorative study with a rather small sample size (*N* = 106, of which 92 participants initiated the intervention and were included in the analysis), resulting in limited statistical power which may partly explain the non-significant findings of the study. Larger studies are needed to be able to assess the impact of automated support on adherence. Second, while this study was one of the first to evaluate the use of a virtual coach in this context, the novelty of the approach may have introduced technical difficulties that may have impacted the use of the intervention. Third, the participant sample was predominantly female and highly educated, which may limit the generalizability of the findings to populations with lower educational backgrounds. Furthermore, many individuals declined participation or did not respond (*n* = 420). A likely factor is that ethical approval information had to be sent by post before enrolment, which may have reduced willingness to participate. Fourth, platform complexity (e.g. the use of two platforms; web-based and smartphone-based) was identified in the qualitative interview as a reason for early dropout, which could have affected the results, as participants struggled with the intervention. Fifth, the analyses regarding motivation and depressive symptoms were conducted only with participants who started the intervention. The results can thus be considered of an exploratory nature only. Sixth, because the intervention lasted four weeks, and the algorithm for the personalized messages required time to learn from user behaviour, it is likely that meaningful personalization only began after the first one to two weeks. As a result, participants may have received just a limited period of truly tailored support, which may not have been sufficient to influence adherence outcomes (Hassouni et al., 2022; Mol et al., 2018). Finally, although we asked participants why they did not complete the intervention, the question was broad and did not explicitly ask about specific components of adherence (e.g. motivational support). This may have limited our ability to draw conclusions about which aspects further affected the adherence.

### 4.4 Future research

Future research should focus on improving the design and functionality of automated support within online interventions. While our findings suggests that automated motivated strategies alone may not be sufficient to improve adherence, their scalability remains a great advantage. More advanced applications of machine learning techniques, such as reinforcement learning and natural language processing, may increase the personalization and responsiveness of support systems. Participants with lower levels of depressive symptoms may already be sufficiently active and motivated, reducing the need for external support to promote adherence. Therefore, future research should consider the suitability of such interventions for different populations. Additionally, future work should look beyond motivation as a factor that influences adherence. Factors such as usability, technical stability, and the perceived effort required to engage with the platform are also critical and should be addressed.

### 4.5 Conclusion

This study explored the potential role of personalized automated motivational support in unguided BA, its overall impact on adherence was limited. Importantly, the limited effectiveness may not lie in the motivational strategies themselves, but in their misalignment with the needs of the specific target population, individuals with relatively mild depressive symptoms and already high intrinsic motivation. For such users, the added value of motivational messages may be minimal, and behavioural activation may offer less room for meaningful change. Additionally, while usability scores fell below the accepted threshold, their direct impact on adherence was not assessed and should be interpreted with caution. Taken together, these findings suggest that future research should consider the suitability of the intervention content and automated support strategies for different symptom severities. Greater attention should also be given to improving platform usability and strengthening personalization within automated support to better meet users' needs in unguided digital mental health interventions.

## Abbreviations

RCT: Randomized controlled trial
ECA: Embodied conversational agent
(i)CBT: (Internet) cognitive behavioural therapy
BA: Behavioural activation
EDC: Electronic data capture
HADS-D: Hospital Anxiety and Depression Scale
SMFL: Short Motivation Feedback List
SUS: System Usability Scale
CSQ-I: Client Satisfaction Questionnaire for internet-based interventions

## CRediT authorship contribution statement

SP, AK and HR wrote the protocol for the study. HR, AK, MC and KA added the third condition to the study. KA and MC coordinated the data collection. KA, MS, AK and HR drafted the manuscript. All authors read and approved the final manuscript.

## Funding information

This study is funded by an EU INTERREG grant for the E-Mental Health Innovation and Transnational Implementation Platform North West Europe (eMen) project. The funder had and has no role in the intervention development, study design, the collection, management, analysis, and interpretation of the data, the writing of the report, or the decision to submit the report for publication.

## Declaration of competing interest

The authors declare no conflict of interest related to this study.

## Figures and Tables

**Table Annex 3:** Comparison of the adherence measures between virtual coach/control condition and between personalized messages/control condition for the total sample (*N* = 106).

Adherence measures	Condition	*N*	*M*^a^	*SD*	(*t*)*df*	*p*	Cohen's d	95 % CI for *d*
Total pages visited (≥15 s)	Control	36	21.5	13.86				
	Virtual coach	35	19.83	14.67	−0.49 (69)	0.665	−0.117	[−0.58, 0.35]
	Personalized messages	35	18.23	13.55	1.0 (69)	0.716	0.239	[−0.23, 0.71]
Number of mood ratings	Control	36	43.67	35.57				
	Virtual coach	35	39.66	41.28	−0.44 (69)	0.612	−0.104	[−0.57, 0.36]
	Personalized messages	35	47.06	66.72	0.61 (69)	0.259	−0.064	[−0.53, 0.40]

a Mean difference scores for the pages visited range from 0 to 55 and for the mood ratings from 0 to 84.

## Annex 1 Overview of personalized motivational messages

### Coaching style: encouragement

Definition: Positive reinforcement of past and future behaviour and motivation to build self-confidence.
Message Title/Icon: “Well done!”/Thumbs-up icon.

**Example Messages:**

1. Great to see that you're still tracking your mood. Good luck with Moodbuster Lite!
2. It's good that you're logging your mood regularly—it helps increase insight into your patterns.
3. Don't give up if you haven't tracked your mood in a while; you can always start again. Keep going!
4. By participating in this course, you can shift your negative cycle into a positive one—go for it!
5. If you're finding it hard to stay motivated, reflect on what you hope to gain from the course.
6. Working with Moodbuster Lite brings you closer to your goals. Great that you're participating!
7. Through Moodbuster Lite, you learn which steps you can take to improve your mood. You're on the right track!
8. Participating in Moodbuster Lite is already a first step towards a better mood!
9. It's commendable that you're making time for yourself to improve your mood. Be proud of that.
10. It can sometimes be difficult to stick with a training like Moodbuster Lite, but you're doing it!
11. Taking this course shows that you value working on your mood. Well done!
12. By participating in Moodbuster Lite, you're giving yourself the chance to work on your symptoms. Good luck!
13. Don't get discouraged when things get tough; have confidence in yourself!
14. It's great that you've started working on improving your mood. Which strengths have you used to make this happen?
15. If you're unsure whether you can address your symptoms, set simple and achievable goals. You can do it!
16. Think about what you've already achieved in this course. You can be proud of yourself!
17. When you're not feeling well, it's sometimes extra hard to work on problems. That you're managing to do so is commendable.
18. It's not always easy to work on problems, but you're doing it! Don't forget to reward yourself occasionally.
19. Changing your behaviour isn't easy, but it's already great that you're working on it!
20. Sticking to your schedule isn't always easy. Reward yourself when it works!
21. Starting a course like Moodbuster Lite is the hardest step! You've already taken that step!
22. You can be proud of yourself and what you're doing to improve your mood.
23. You deserve to reward yourself when you regularly track your mood.
24. You can be satisfied with your efforts in Moodbuster Lite, even when not everything goes perfectly!
25. Remember that not only you benefit from this course, but also everyone around you!
26. By following this course, you've already taken the most important step!
27. Moodbuster Lite is a tool; the real work is done by you!
28. It's great that you're tracking your mood again; this can truly bring about change in your life!
29. Following this program is impressive! Keep it up!
30. Following Moodbuster Lite requires discipline, something you can be proud of!
31. You're already heading in the right direction by tracking your mood daily. Keep it up!
32. Trying to improve your mood comes with ups and downs. When did you experience an ‘up’ moment this week?
33. It's unrealistic to expect only upward progress; being engaged in the process is already valuable!
34. Taking some time for yourself when you track your mood—link this to something that makes you feel good!
35. The time you dedicate to Moodbuster Lite is ultimately time you give to your future self! Keep it up!
36. This program teaches you a lot about your own mood. You're doing well!
37. Focusing on your own mood can sometimes be challenging. Trust the process!
38. It's great that you're active in Moodbuster Lite. What did you see or experience today that made you happy?
39. Hold on to positive thoughts; you're doing a fantastic job!
40. A setback happens to everyone; engaging with Moodbuster Lite is another step in the right direction!

### Coaching style: information

Definition: Messages that refer to specific platform features or suggest next steps.
Message Title/Icon: “Tip!”/Lightbulb icon.

**Example Messages:**

1. In the mood chart, you can accurately see the progression of your mood.
2. In the mood chart, you can track the effect of the course on your mood.
3. You can use the agenda function to register and evaluate activities.
4. On the homepage, you can see what you were working on the last time you visited Moodbuster Lite.
5. Reminders can help you not forget enjoyable activities.
6. In the agenda, you can keep track of which enjoyable activities you do and how your mood is at that moment.
7. Writing down how you felt during enjoyable activities is a good way to choose activities that suit you best.
8. Did you know that you can always view and edit completed exercises?
9. If you find it difficult to come up with enjoyable activities, you can find a list of suggestions in exercise 1 of this lesson.
10. In your agenda, you can look back at activities you planned in the past.
11. Forgot which enjoyable activities you can do? You can always read your notes on the website.
12. It's sometimes useful to reread the training content to refresh your knowledge.
13. By formulating course and life goals, you give direction to the course.
14. Formulating course and life goals isn't always easy; in the introductory lesson, you'll find some tips.
15. The ‘help’ function provides more information on what to do when your symptoms worsen.
16. If you have technical questions about Moodbuster Lite, the ‘help’ function might assist you.
17. You can see an overview of all completed exercises under ‘exercises’.
18. It's good to alternate between necessary and enjoyable activities.
19. If you find it difficult to come up with rewards, the ‘psycho-education’ lesson offers some tips.
20. The videos in the course provide a brief introduction to the focus of each lesson.
21. The exercises in the course help to apply theory to practice, tailored to your own situation.
22. In the app, you can change the display of mood registrations by clicking on the graph icon.

### Coaching style: affirmation

Definition: Shows understanding and empathy to validate feelings, thoughts, and behaviours.
Message Title/Icon: “You're not alone”/Empathy icon.

**Example Messages:**

1. It's normal not to feel cheerful all the time—don't worry if that happens.
2. Many people feel low sometimes. It's nothing to be ashamed of.
3. Feeling tired often? That can easily happen when you're following a course like Moodbuster Lite!
4. It can be challenging to constantly think about how you feel—that's understandable.
5. Don't worry if you forget to track your mood; it's impossible to remember every time.
6. It can be hard to always keep up with mood tracking—that's completely normal!
7. When you're not feeling well, it's extra difficult to track your mood; don't be too hard on yourself during these times!
8. Don't get discouraged if you sometimes forget to track your mood—that happens to the best of us.
9. Participating in this training can be quite complex; it's okay if you sometimes forget to follow it.
10. Don't let it get you down if you can't carry out your planned activities—that happens to everyone sometimes.
11. Everyone struggles to find time to follow the course; that's completely normal, so don't worry if it happens to you.
12. It's completely normal to sometimes feel unmotivated about participating in the course; don't worry if you experience this.
13. Carrying out planned activities isn't always easy; it's okay if it doesn't work out immediately.
14. Don't worry if you don't achieve course goals right away; for most people, it takes time before they see progress.
15. Most people find it hard to stick to a schedule; it's okay if that's the case for you too.
16. It's difficult for many people to change their situation; it's okay if it doesn't happen immediately.
17. It can sometimes be challenging to keep the end result in mind during the process; know that the process itself is already educational.
18. It's okay if you don't feel motivated to do things for a while; accept this and take a moment to rest.
19. Sometimes it feels like everything is going wrong—that's normal and okay.
20. Sometimes getting up and doing small tasks is already exhausting; many people experience this, and that's okay.
21. It's okay to feel lonely in your process sometimes; many people experience this.
22. Sometimes it feels like you're on your own, and that's really tough; it's okay to feel down about this.
23. Feeling down is completely normal and happens to everyone; don't worry too much if it happens to you.
24. Moodbuster Lite may sometimes feel challenging, and that's okay—you don't always have to be positive about everything.
25. Sometimes you might have negative thoughts that seem to take over; try not to suppress them all but remember that this is temporary.
26. You don't have to suppress negative emotions; everyone experiences them sometimes, and you can accept them too.
27. It can be really challenging to always be active in Moodbuster Lite; it's okay if this feels difficult.
28. Maybe it's sometimes hard to remember that Moodbuster Lite is there to help you; it's okay to remind yourself of this occasionally.
29. Feeling down for a few days is really unpleasant; remember that this is temporary and allow yourself that time.
30. It can sometimes feel quite challenging to always be dealing with your feelings; this is very understandable.

## Annex 2 Participant-reported reasons for non-adherence

VC: Virtual Coach
AM: Automated Messages
C: Control.

### 1 Complexity of using the platform

1. I didn't know when to start the last part, so by the time I began, the four weeks had already passed. (VC)
2. Confusion about the website. (C)
3. Misunderstanding on my part. (AM)
4. Didn't get around to it, it was just a bit too much effort to go to the website (a pop-up reminder via the app, like for mood tracking, could help). (C)
5. I didn't fully understand the order in which I was supposed to continue. (C)
6. I think I still have one lesson left?
7. Because I wasn't even able to get started.
8. Login problems. (C)
9. Because I couldn't log in to complete the final lesson. (VC)

### 2 Confronting

1. Too confronting. (AM)
2. After a few days, I dismissed the mood tracking notifications—I felt like they only emphasized how bad I was feeling. (VC)

### 3 Time-consuming

1. Unfortunately, I had a severe case of COVID, so I missed part of the program. I was very ill and felt miserable. I'm doing better now. I'm sorry I couldn't complete everything. (AM)
2. I'll still finish it, but I started a bit late.
3. I apparently missed the course entirely. I did ask about it once. I only managed to log my mood but didn't get anything else out of it.
4. Lack of time. (C)
5. Takes too much time, offers little added value. (C)
6. Hard to apply with a busy schedule. (C)
7. I was very busy during this period and couldn't always concentrate on it. (VC)
8. Too much hassle. (AM)
9. Time shortage. (C)
10. Work pressure, fatigue, concentration problems due to tapering off medication. (AM)

### 4 Not helpful

1. I didn't get anything out of it, didn't like the assignments, and found the agenda entries awful haha. (AM)
2. Felt the course didn't match my situation. (C)
3. I missed the personal contact and interaction. (C)
4. I found the questions very general and meaningless. (VC)
